# Memory Control: A Fundamental Mechanism of Emotion Regulation

**DOI:** 10.1016/j.tics.2018.07.015

**Published:** 2018-11

**Authors:** Haakon G. Engen, Michael C. Anderson

**Affiliations:** 1 Medical Research Council (MRC) Cognitive and Brain Sciences Unit, University of Cambridge, Cambridge, UK; 2 Neuroimaging Center, University Medicine Mainz, Mainz, Germany

**Keywords:** emotion regulation, memory control, inhibition, direct suppression, thought substitution

## Abstract

Memories play a ubiquitous role in our emotional lives, both causing vivid emotional experiences in their own right and imbuing perception of the external world with emotional significance. Controlling the emotional impact of memories therefore poses a major emotion-regulation challenge, suggesting that there might be a hitherto unexplored link between the neurocognitive mechanisms underlying memory control (MC) and emotion regulation. We present here a theoretical account of how the mechanisms of MC constitute core component processes of cognitive emotion regulation (CER), and how this observation may help to understand its basic mechanisms and their disruption in psychiatric disorders.

## Highlights

The neural correlates of emotion regulation have been studied extensively over the past decade, but without consideration of the mechanistic role of memory control.

Knowledge of the neurocognitive mechanisms enabling the suppression of memories and thoughts from conscious awareness has grown increasingly specific.

Recent studies reveal an association between memory control deficits and affective psychopathologies such as depression and post-traumatic stress disorder, suggesting that controlling memory and affect are interrelated.

Successfully controlling the retrieval of intrusive memories reduces their later emotional impact via modulation of amygdala activity, indicating that controlling memories regulates affect.

Memory control processes may provide a mechanistic foundation for emotion regulation, thereby contributing to a unified account of the mechanisms underlying this process.

## Memory Is Central to Affective Experience

Affective science has often sought to explain how external stimuli cause emotional reactions [1]. Following from this, research on emotion regulation has emphasized (with notable exceptions 2, 3, 4) the mechanisms that enable us to pursue our goals in the face of external emotional stressors [5]. Understanding how people regulate **exogenously elicited emotions** (see Glossary) is, and should be, a fundamental goal of emotion regulation research. However, research on emotion regulation would benefit from a mechanistic understanding of an equally great – perhaps even greater – source of emotional states in our daily lives: memory. Outside the immediate experience of emotional events, any later impact – adaptive or maladaptive – that they have on our lives is mediated by memory. Like Proust’s *madeleines*, the triggers for this are often stimuli that most people would consider innocuous, but that by virtue of evoking an episodic memory in the individual have the power to elicit neural, physiological, and subjective reactions mimicking those of the original experience 6, 7. Thus, people can **ruminate** on past emotionally significant events, frequently leading to the reinstatement and perpetuation of (mostly negative) emotions from their past. Similarly, when people worry about their futures, they engage **episodic prospection** processes that can engender **endogenous emotional reactions** even though the eliciting events have not occurred [8]. Such self-generated emotional states can be a major source of stress and are thought to play a central role in the etiology of psychopathology 9, 10. Indeed, this is likely the case even for disorders primarily characterized by aberrant reactions to stimuli, because even exogenously elicited emotions are robustly influenced by memory: emotional experiences are not mere reactions to the environment but are caused by both the properties of the stimulus and the context in which it occurs 11, 12, 13, 14, 15. This context consists not only of physical factors (e.g., whether one encounters a snake in a terrarium or on a forest path) but also one’s history with a stimulus and the expectations thus engendered; expectations that are rooted in internal **predictive models** 16, 17, which are themselves grounded in memories [18]. Consequently, if it were possible to modulate the accessibility of memories, one could change either exogenously or endogenously generated emotional responses – in effect, regulating emotion 1, 19. Despite these illustrations of the important role of memories in shaping our emotional lives, how we regulate emotions by downregulating the memory traces that contribute to them has attracted comparatively little attention in research on emotion regulation.

## Memory Control Is Fundamental to Cognitive Emotion Regulation

In this Opinion article we outline a view of the mechanisms supporting the regulation of **mnemonic emotional material** building on emerging findings from research on **memory control** (MC). Our starting point is the observation that, because our emotions are frequently driven by memories, controlling the accessibility of such memories to awareness should be an effective way of regulating one’s emotional reactions [20]. Nevertheless, various factors have contributed to the view that attempts to voluntarily suppress thoughts and memories are unhelpful, and are perhaps harmful (Box 1). Contrary to this, we claim that controlling the occurrence and/or constitution of an upsetting memory – in short, MC – is the ultimate objective of successful emotion regulation of endogenous materials.

Box 1 **Differentiating Direct Retrieval Suppression from Other Suppressive Phenomena**

A potential source of confusion about our argument is the tendency to equate DS with other, often counterproductive, suppressive phenomena. For example, in emotion regulation research ‘suppression’ usually refers to **expressive suppression** (ES), which involves inhibiting the behavioral expressions associated with emotional states (e.g., adopting a poker face to hide emotions). Owing to its small or even paradoxical effects on subjective emotion 74, 85, and because the trait tendency to use ES is associated with poor mental health outcomes [86], ES is often considered to be a maladaptive emotion-regulation strategy. However, DS clearly differs from ES in that it directly acts on the representation of the unwanted memory, rendering it less accessible. As such, DS is a fully cognitive strategy, involving different regulatory mechanisms.

Another related line of research is thought suppression, as explored through the ‘white bear effect’. In this research participants are instructed to not think of a specific item (e.g., a white bear) over a period of 5 minutes, and report when it comes to mind [87]. Research using this paradigm has shown that participants have difficulty avoiding thinking about the item, and this has been taken to show the inefficacy and counterproductive nature of thought suppression 87, 88. While DS conceptually resembles the ‘white bear’ variety of thought suppression, the experimental procedure used to investigate the latter has characteristics that make suppression inherently unlikely to succeed. Specifically, the white bear task makes explicit reference to a specific forbidden thought to be suppressed. However, achieving this stated goal is effectively impossible because simply remembering the purpose of the task requires that participants violate the task goal. This contrasts with the DS task, which does not integrate the specific memory/thought to be avoided as part of the task goal, rendering retrieval suppression possible. This ‘goal-integration theory’ may account for the apparent discrepancy between work on MC and the Wegner thought-suppression paradigm [89], and suggests that the white bear task might fail to capture the true utility of suppressive processes measured in work on MC. Consistent with this, a meta-analysis [90] of 33 studies of psychopathology using the white bear task found no differences in suppression success or in rebound effects across patients and controls, raising concerns about whether this the task captures the mechanistic deficits that lead to intrusions in real life.

Researchers have also investigated the relationship between thought suppression and psychopathology via the white bear suppression inventory (WBSI). Such work has contributed to the widely repeated conclusion that suppression, as a coping strategy, is associated with increased risk of psychopathology [91]. Although the WBSI was intended to measure tendency to suppress thoughts, large-scale studies have now repeatedly found that the scale measures both the tendency to suppress thoughts and the degree to which they fail at doing so 92, 93, 94. Importantly, failure at, but not tendency to, suppress thoughts predicts psychopathology. Indeed, when successful thought-control ability is separately quantified [41] it robustly predicts reduced symptoms of anxiety, depression, and other conditions characterized by intrusive symptomatology. These findings are consistent with the possibility that, in normative samples, thought suppression may play an important beneficial role in mental health.

Alt-text: Box 1

More controversially perhaps, we further propose that MC mechanisms underlie the benefits of many of the **volitional cognitive emotion regulation** (CER) phenomena that have been identified, even when regulation is targeted at exogenously driven emotional reactions. Specifically, we claim that CER strategies can be classified by whether they employ two core mechanisms of MC that reduce access to memory traces that contribute to emotional responses. Importantly, we propose that the mechanisms of MC offer a novel account of how emotion regulation is implemented, that may help to understand its normal functioning and also psychopathological syndromes. This proposal is motivated by growing evidence that instructed MC abilities are related to beneficial outcomes (reviewed below) and the belief that similar mechanisms may support **spontaneous MC**, and therefore **spontaneous CER**, although further research will be necessary to test this assumption. As such, we propose that MC is crucial to regulating both mnemonically and exogenously elicited emotional states, and should be considered to be a fundamental process of volitional emotion regulation.

## Memory Control Enables Regulation of Contents of Thought

MC can be defined as the capacity to volitionally influence the contents of thought in a goal-directed fashion by reducing the accessibility of memories [20]. MC is often exerted reactively, in response to reminders that trigger the automatic retrieval of an unwanted memory or thought. Two mechanisms have been identified that enable MC when someone confronts an unwelcome reminder: (i) **direct suppression** (DS), involving the stopping or cancellation of the episodic retrieval process initiated by the cue, and the inhibition of the unwanted trace, and (ii) **thought substitution** (TS), involving the engagement of episodic retrieval, but instead being redirected towards alternative memories to occupy the limited focus of awareness, and to inhibit the to-be-avoided memory [20]. Both of these mechanisms can be voluntarily deployed, and both induce forgetting of unwanted memories 21, 22, 23, 24, enabling motivated forgetting of both neutral and affectively charged memories 20, 24, 25, 26. Crucially, because these processes allow a person to avoid awareness of memories, they are sometimes mistakenly equated with other forms of **cognitive avoidance** that are often associated with poor psychological outcomes [27]. The capacity to induce forgetting sets these mechanisms apart from conventional cognitive or behavioral avoidance: MC involves not simply avoiding reminders that trigger unwanted thoughts or memories but instead involves directly confronting those reminders and actively altering our cognitive response to them, and reducing the accessibility of the associated memory trace.

### Direct Suppression of Retrieval

**Figure 1:**
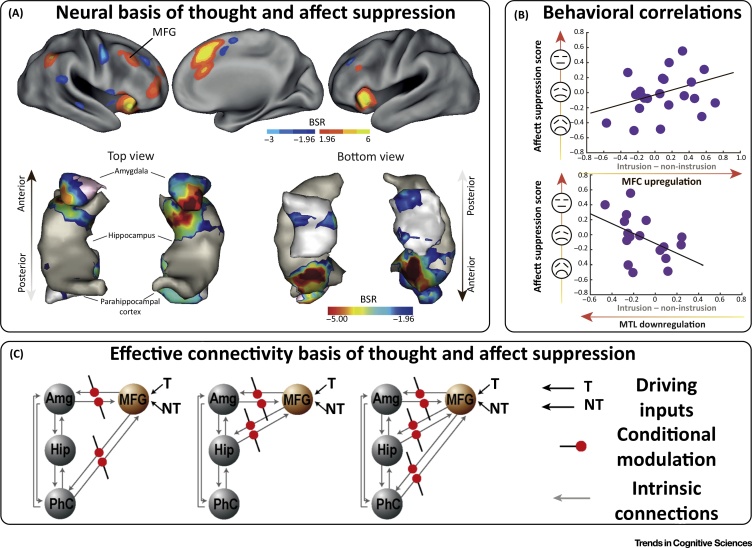
Neurobehavioral Markers of Affect Suppression Following Retrieval Suppression. Summary of results from [26] (adapted with permission). This study showed that DS both reduced the intrusiveness of affective images and had a lasting impact on affective reactions to them, such that subjective evaluations of suppressed stimuli were less negative. This **affect-suppression effect** resembles **extinction learning**[105], where affective responses to a conditioned threat-signaling stimulus are downregulated by repeated experiences that it no longer signals threat. This similarity also extends to the neural domain, and data suggest that direct suppression (DS) may engage prefrontal circuits to increase activity in inhibitory GABAergic interneurons within the MTL 58, 60, whereas extinction learning occurs via prefrontally mediated engagement of GABAergic inhibitory circuits within the amygdala 105, 106. This suggests that the affective consequences of suppression could also rely on similar downregulation of the amygdala, a notion supported by analyses [23] showing that DS was associated with upregulation of prefrontal circuits and downregulation of the amygdala when aversive images intruded into awareness and needed to be purged (A). Importantly, the strength of this downregulation was associated with larger affect-suppression effects and fewer involuntary intrusions, indicating that that these neural effects were key to successful mnemonic and affective control (B), with effective connectivity analyses demonstrating that suppression effects were driven by the right MFG, which effected parallel suppression of the amygdala, the hippocampus, and the parahippocampus (C). It is unknown how the MFG achieves these suppression effects because MFG is not directly connected to either the amygdala or the hippocampus. However, it is connected to several regions that are thought to implement amygdala regulation, including the ventrolateral prefrontal cortex (vlPFC) 66, 67, 68, 107, and dorsal 107, 108 anterior cingulate 105, 107, 108, 109. These regions are also consistently engaged in DS (panel A; also Figure 2A and [59]), suggesting that they might be intermediate elements of a top-down regulatory pathway. Abbreviations: Amg, amygdala; BSR, bootstrapped standard ratio; Hip, hippocampus; MFG, mid-frontal gyrus; MTL, medial temporal lobe; NT, no-think; T, think.

DS of retrieval should not be confused with other forms of suppression that are often discussed in research on emotion regulation, and which have been argued to be ineffective and maladaptive (Box 1). Extensive evidence shows that suppressing retrieval in response to reminders is possible, and that doing so has persisting effects on the accessibility of suppressed traces 21, 25, 28, 29, 30, 31, 32, 33, 34, 35. DS begins when one encounters a reminder to an unwelcome memory or a thought. Such reminders are believed to trigger (owing to the affective qualities of the reminder, or of the unwelcome memory) inhibitory control to countermand the retrieval of the associated event, in a manner analogous to the way that control processes countermand motor processes to stop physical actions. Evidence indicates that repeated DS over multiple encounters with a reminder weakens the associated memory until it no longer intrudes 26, 32, 36, 37, ultimately impairing voluntary retrieval of the memory, consistent with an active memory-inhibition mechanism [38]. Of course, memories vary in how amenable they are to suppression, and individuals vary in their ability to implement such suppression [29]. Importantly, impaired DS is related to both subclinical traits associated with affective psychopathology (e.g., rumination [39] and worry [40]), as well as pathology itself (e.g., **post-traumatic stress disorder,** PTSD [41], and depression [42]). Crucially, DS of aversive images can reduce one’s affective evaluation of those images at a later time (Figure 1) [26]. Interestingly, similar long-term effects arise for the CER strategy of **reappraisal** [43], raising the possibility that reappraisal engages regulatory mechanisms similarly to DS.

### Thought Substitution

One can also prevent recall of unwanted memories by diverting the retrieval process and recalling a substitute memory instead, preferably one that is innocuous or even positive 25, 44. Such TS may be especially useful when reminders are sufficiently powerful to make DS difficult, such as with traumatic memories [45] or ruminative topics [46]. Compared to DS, there has been less research on TS, but evidence suggests it is at least as effective as DS at causing voluntary forgetting 25, 47, 48. Interestingly, TS appears to cause forgetting even for individuals with impaired DS, such as those suffering from major depression [49], suggesting that they are supported by distinct mechanisms. In the context of CER, there is a conceptual resemblance between TS and the CER strategy of distraction 43, 50, 51. Although distraction can involve TS, distraction is a more general strategy that involves any reorienting of attention away from emotional stimuli to occupy awareness with non-emotional materials 51, 52. Although TS has a similar goal, it specifically involves the retrieval of alternative memories in response to the unwelcome reminders rather than merely a reorienting of attention. This generation process not only encodes an alternative path for retrieval, to follow in the future when that reminder is encountered, but also can inhibit the original memory, possibly by the mechanisms of retrieval-induced forgetting 53, 54. Thus, TS trains access to non-dominant associations for a given stimulus, in addition to occupying immediate awareness with distraction. For instance, if, upon seeing one’s ex-partner, one is reminded of upsetting memories about one’s lost love, one might instead recall the good times with the person, or even generate associations to new thoughts of a current, hopefully happier, love interest, thereby transforming one’s emotional reactions to the person 55, 56, 57. As we shall see later, this is also reminiscent of the cognitive operations supporting the CER strategy of reappraisal which, like TS, often entails the generation of an alternative representation of an unwanted percept or memory.

## Neural Architectures of Direct Suppression and Thought Substitution

**Figure 2:**
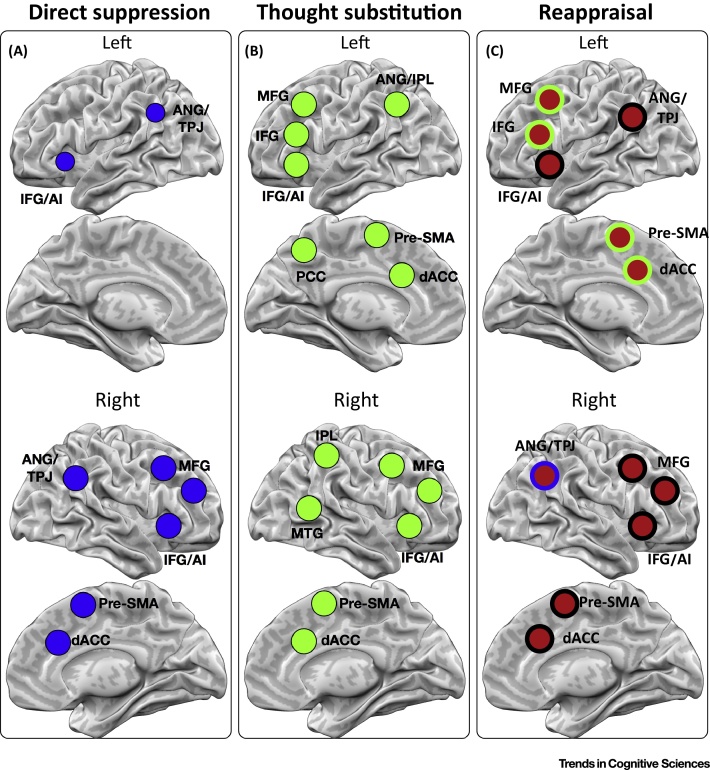
Neural Networks Underlying Memory Control (MC) and Reappraisal. Networks for (A) direct suppression, adapted from [59]; (B) thought substitution, adapted from [25]; and (C) reappraisal, reproduced, with permission, from 71, 72. Reappraisal areas are color-coded according to overlap with MC circuits. Blue outline, retrieval suppression; green outline, thought substitution; black outline, both. Abbreviations: AI, anterior insula; ANG, angular gyrus; dACC, dorsal anterior cingulate cortex; IFG, inferior frontal gyrus; IPL, inferior parietal lobule; MFG, mid-frontal gyrus; PCC, posterior cingulate cortex; SMA, supplementary motor area; TPJ, temporoparietal junction.

DS and TS achieve forgetting of unwanted memories by distinct although partially overlapping neural mechanisms (Figure 1; 20, 58 for detailed discussion). DS engages what may be a domain-general inhibitory control mechanism supported by the (primarily right) dorsolateral prefrontal cortex (dlPFC) 34, 59 that, in the context of retrieval suppression, dynamically interacts with the **hippocampus** to suppress its activity and disrupt retrieval 25, 26, 32 (Figure 2A). This suppressive effect is especially pronounced when memories involuntarily intrude into awareness and need to be purged 36, 37. The pathway by which hippocampal activity is suppressed by prefrontal regions is presently unclear, although primate neuroanatomy suggests that it could involve anterior cingulate cortex (ACC) circuits central to top-down regulation [58]. Whatever the pathway may be, evidence indicates that the concentration of γ-aminobutyric acid (GABA) in the hippocampus predicts individual differences in how effectively one forgets via DS [60], suggesting that **GABAergic interneurons** implement the proximate mechanism that suppresses activity in the hippocampus, and, by extension, supports DS. Interestingly, this suppressive mechanism appears to have a widespread effect on the retention of all recent events that depend on hippocampal activity for stabilization, inducing an **amnesic shadow** for events in the period before and after suppression [61]. TS, by contrast, does not cause this shadow [61], again suggesting that its mechanisms are distinct. Because suppression reduces intrusions over repeated encounters with reminders, the need for prefrontal control declines adaptively, and more so for individuals who are better at suppression [32].

Suppressing intrusive thoughts is not solely about modulating hippocampal activity and explicit memory, however. An important feature of DS is that it also suppresses regions outside the hippocampus that are involved in representing the specific content of a given memory, including, for example, fusiform cortex and parahippocampal cortex for objects and places, respectively [62]. Correspondingly, DS disrupts implicit expressions of memory such as perceptual priming that are believed to rely on these neocortical representations 62, 63, 64. The need for inhibitory control to target both the hippocampus and neocortical regions is thought to arise because the unwelcome reminder elicits pattern completion of a memory in the hippocampus, in turn triggering re-entrant activation of cortical regions representing mnemonic content 26, 62, 64. Suppressing awareness, consequently, entails parallel targeting of inhibition in neocortical regions expressing this onset of activity. This **reinstatement principle** extends to emotional memories: during memory intrusions, DS of aversive scenes reduces activity in the **amygdala** and vmPFC [26] – structures that are closely associated with the generation of subjective affect 7, 56, 65. Importantly, DS of memories of aversive scenes attenuates negative emotional judgments about those scenes when they are later encountered (Figure 1). This suggests that MC mechanisms, first identified outside the context of emotion regulation using purely neutral stimuli, can be deployed both to suppress mnemonic traces of stimuli as well as to directly suppress the affective connotations of the stimuli themselves. Indeed, this suggests that endogenously focused CER can be seen as a special case of the general reinstatement principle introduced above, such that suppression of affective connotations is a direct consequence of stopping the retrieval and neural reinstatement of affective mnemonic traces. If this account is correct, DS of affective material will, in effect, be an act of emotion regulation.

In contrast to the strong right-lateralized activations observed for DS, TS is predominantly left-lateralized, centered on caudal prefrontal (cPFC) and ventrolateral prefrontal (vlPFC) cortex (Figure 2). The vlPFC supports selective retrieval, especially under conditions of high retrieval competition 25, 66, and coupling between cPFC and vlPFC predicts the amount of forgetting elicited by TS [25]. Whereas the functional significance of this coupling remains unknown, one plausible interpretation is that cPFC entrains vlPFC to drive retrieval of alternative memories associated with the cue to supplant the dominant, to-be-avoided, memory. Supporting this, TS does not reduce hippocampal activity but can even increase it, especially under conditions of high competition [25], suggesting that the vlPFC interacts with the hippocampus to facilitate retrieval of the non-dominant memory. The pathway underlying this prefrontal–hippocampal interaction during TS has not been established, but related data suggest that it may take place via the uncinate fasciculus, which has been shown in humans and non-human primates to link these regions to the anterior hippocampus and to be related to controlled semantic retrieval 67, 68, 69.

Taken together, these findings indicate that TS and DS constitute distinct routes to achieving MC, supported by distinct cortical networks 25, 26 that exert opposing influences on hippocampal activity and different functional aftereffects [61]. Importantly, the fact that they appear to be supported by separable mechanisms suggests that it may be possible to exert superior control over memories by using DS and TS in conjunction [70]. In the following section we argue that this may be what occurs when people engage in CER using the ‘gold-standard’ emotion-regulation strategy of reappraisal.

## Direct Suppression and Thought Substitution for Reappraisal

Reappraisal is by far the most-studied CER technique, and has been shown to be effective at both reducing negative and increasing positive emotion [71]. Reappraisal involves an effort to re-evaluate a stimulus to alter its emotional impact 5, 72, 73, 74, most commonly by changing the narrative associated with a stimulus 73, 75. For instance, when confronted with blood and gore evoking strong visceral reactions of disgust, one can remind oneself that one is in a movie theatre watching a horror film, implying that the distressing images are almost certainly fake. Alternatively, if one happens to want the full horror-film experience, one might suppress thoughts of being in a cinema and instead focus on the narrative portrayal that led to the carnage, which would likely amplify one’s emotional reactions. Thus, by accessing different aspects of the information about an emotional event, one can alter its interpretation, reducing or increasing its emotional impact. Notably, while most frequently studied in the context of exogenous emotional reactions, reappraisal can be used to regulate both stimulus-based and memory-based emotional reactions 2, 3, 76, 77, meaning that it has a similar remit to MC.

Mechanistically, reappraisal is best viewed as a complex process supported by multiple cognitive functions [78]. Although there are many reasons why reappraisal might succeed in regulating emotional responses, we suggest that a key reason is that it relies upon the two MC mechanisms discussed above to reduce the current and future likelihood of retrieving representations of distressing interpretations. Specifically, we suggest that, as the reappraisal process unfolds, it involves (i) the initial DS of the representation of the primary affective narrative, and (ii) the generation of a substitute narrative in response to the stimulus, constituting a clear case of TS. Importantly, we suggest that this underlies not only simple reappraisals that may interfere with retrieval of primary emotional narrative but also more elaborate reappraisals that transform the meaning of the emotional event itself. Thus, for instance, in the preceding example in which emotional reactions to a horror movie were regulated, reappraisal plausibly involves (i) suppressing the primary emotional narrative of the external stimuli. and (ii) focusing on details of the scene or context that enable search of memory for an alternative interpretation (e.g., the blood is only ketchup) that can then substitute the primary narrative. Both of these are MC operations, and together they should reduce awareness of the immediate aversive interpretation and orient affective responses towards a more innocuous narrative as reappraisal takes place.

A key finding distinguishing reappraisal from other forms of CER is that it has lasting effects on subjective responses to affective stimuli [43]. This is explained by the current account because TS associates novel information generated during the reinterpretation process with the stimulus or memory. Thus, subsequent presentations of the stimulus should also evoke retrieval of the alternative interpretation, in the same way as substitute thoughts block the retrieval of unwanted memories in studies of MC [25]. We argue, therefore, that the lasting effects of reappraisal on affective responses to stimuli or to memories can be properly regarded as motivated forgetting achieved via MC, possibly leveraging fundamental affective memory updating phenomena such as reconsolidation (Box 2).

Box 2 **Reconsolidation, Emotion Regulation, and Memory Control**

A phenomenon that has received increasing attention as a way to enact memory-based emotion regulation is reconsolidation 95, 96, 97. Research on reconsolidation suggests that, when both emotional and non-emotional memories are retrieved, they exhibit increased lability, leaving the memory trace vulnerable to interference. This retrieval-induced plasticity is thought to be important for enabling old memories to be updated by new information, but comes at the cost that retrieved memories require restabilization or reconsolidation to be available for retrieval at a later date [98]. By interfering with this reconsolidation process using behavioral or psychopharmacological interventions, evidence suggests that it is possible to erase memory traces, including emotional threat memories in humans [99]. Moreover, in the lability period it is also possible to update or alter memories. For example, a negative memory may be changed to be less negative by integrating new information, as is seen in extinction, a process that has been argued to be central to reappraisal [100], and arguably to CER efforts in general, to the degree that they have lasting changes on the affective reactions subsequently elicited by a memory.

The conditions leading to forgetting in research on MC resemble those thought to be crucial for reconsolidation phenomena. For instance, like reconsolidation interference, MC also involves altering the retrieval process, with both phenomena eliciting forgetting. Inhibitory control may act like an endogenous ‘amnestic agent’ that disrupts reconsolidation. Interestingly, suppression-induced forgetting (SIF) arising via DS has been linked to actively inhibiting the memory from awareness when it intrudes 26, 36, indicating that the memory has already been (at least partially) retrieved. This suggests that SIF could involve interfering with the reconsolidation of memory traces. Similarly, SIF produced by TS (at least as we propose it to be employed during reappraisal) might involve reconsolidation mechanisms to ‘overwrite’ a primary emotional narrative. In line with this, it has been shown that reappraisal, but not distraction, has durable effects on neural reactions to emotional stimuli [43], albeit in the context of what is effectively consolidation of emotional memories following initial presentation and regulation of reactions to emotional stimuli. Because memory disruption effects are observed immediately, it is clear that mechanisms other than reconsolidation are also at play in memory inhibition phenomena. Nevertheless, for CER of endogenous emotional reactions, it is plausible that reconsolidation interference and updating also play a role, although specific research to explore this topic is needed.

Alt-text: Box 2

## Component Process Architecture of Reappraisal and Memory Control

If our MC hypothesis of reappraisal is correct, reappraisal should recruit the neural systems supporting DS and TS. Figure 2 summarizes evidence on the neural bases of DS [59], TS [25], and reappraisal 71, 72. Crucially, the neural correlates of DS and TS were identified via simple paradigms involving MC of neutral stimuli, often simple word pairs [59]. These methods were designed to isolate the fundamental mechanisms that enable a person to stop or redirect episodic retrieval. As such, overlap with reappraisal can be interpreted parsimoniously as the latter process engaging fundamental MC mechanisms. Figure 2C illustrates the overlap between the neural systems involved in reappraisal and DS (blue outline), TS (green outline), or both DS and TS (black outline), demonstrating that the brain regions engaged during these processes overlap considerably, with all major nodes of the reappraisal network being part of the MC networks. Notably, left lateral nodes of the reappraisal network are predominantly associated with TS, with the exception of inferior frontal/anterior insula, as well as the parietal nodes, which are also associated with DS. In the right hemisphere, there is extensive overlap between TS and DS, as well as with reappraisal nodes. This could indicate a hitherto unexplored involvement of DS processes in TS, plausibly related to an initial suppression of recall to better implement TS, similar to the role we propose for DS in reappraisal. Taken together, these observations are consistent with the proposal that the mechanisms supporting DS and TS constitute distinct component processes of reappraisal. It must be made clear, however, that this is a reverse inference; future work will need to experimentally manipulate the putative involvement of TS and DS in reappraisal and relate these findings to TS and DS abilities in more conventional MC tasks. If our account is correct, one would expect DS ability to be associated with the capacity to use reappraisal to inhibit emotional reactions, but not necessarily to the capacity to supplant them with alternative interpretations (as in e.g., positive reappraisal 79, 80), which should instead be correlated with TS performance.

## CER Deficiencies: A Problem of MC?

Beyond its role in reappraisal, a link between MC and CER more broadly is suggested by the strong association between perseverative cognition and affective psychopathologies. Although psychopathologies cannot be reduced to single-factor explanations, they are frequently related to difficulties regulating emotional states elicited by memories or thoughts. The clearest examples of this come from anxiety disorders – that tend to be characterized either by worry about the future, or, as in the case of PTSD, by overwhelming emotional memories of past events. Indeed, both anxious worry [81] and PTSD [41] have been associated with MC deficits. Moreover, it has been shown that suppressing future worries engages MC networks to reduce activation in the hippocampus, amygdala, and vmPFC, presumably to interrupt episodic prospection [40]. Importantly, the capacity to reduce apprehensiveness about feared events via suppression was modulated by trait levels of anxiety, supporting the notion that MC deficits play a part in anxious symptomatology. Similarly, depression is associated with elevated rumination, a problem of persisting recurring thoughts or memories that occur in a context-inappropriate fashion [46]. Rumination is thought to be caused by an inability to disengage from endogenously generated negative autobiographical memories [46], raising the possibility that depression is associated with MC difficulties. As this observation suggests, depression is associated with both neural and behavioral abnormalities in MC [42], and the evidence also indicates that training MC might be an effective (adjunct) treatment for managing depression 49, 70. Conversely, good trait MC may impart **resilience** to emotional stressors, as seen in a recent study showing that behavioral and electrophysiological metrics of DS predict the number of intrusions experienced by participants in the week following viewing of a strongly negative film [82]. Similarly, the ability to engage in motivated forgetting using DS is related to the ability to forgive others for their transgressions [83]. Experience of contending with intrusive memories may also improve MC and future resilience: in healthy samples, retrieval suppression ability increases with the degree of adversity someone has experienced [84]. Thus, at least for DS, the evidence suggests a broader link between MC abilities and the capacity to limit the impact of negative events on one's affective constitution, which could be conducive both to CER and to resilience.

## Viewing Cognitive Emotion Regulation as Memory Control Suggests Novel Mechanisms

Adopting a MC perspective may improve our understanding of the neurocognitive mechanisms supporting CER in general. Although a strong case can be made for thinking of reappraisal as a MC phenomenon, generalizing this proposal to CER strategies more broadly is difficult owing to lack of data on other regulation strategies. For example, a recent meta-analysis found that fivefold more neuroimaging experiments had studied reappraisal versus all the other strategies combined [71]. This problem is compounded by the wide variety of CER strategies proposed, with little agreement on protocols for studying them, much less for comparing their mechanisms of action.

One approach to exploiting what is known about the architecture of MC would be to characterize CER strategies according to the involvement of MC mechanisms. In Table 1 we offer an initial conjecture on how MC might play a role in a range of common emotion-regulation strategies (adapted from [27]), suggesting potential contributions of DS and TS to each. This list is not intended to be exhaustive, but instead aims to provide some initial testable hypotheses: if, as we suggest, MC processes contribute to emotion regulation, individual differences in DS and TS ability should predict the capacity and/or preference for deploying different CER techniques. At present, it is unknown how strongly correlated DS and TS abilities are, but, given the partially dissociable neural systems underlying each, and given the fact that TS may be easier for people deficient in inhibitory control, the ability to wield these mechanisms may be at least partially distinct. Individual differences in DS and TS should predict differing engagement of their respective brain networks during reappraisal. Thus, by investigating reappraisal implementations [73] and other forms of CER (Table 1) that could engage MC processes, the proposed role of the MC architecture could be tested. Moreover, as discussed above, efficacy at implementing DS or TS should predict the specific implementations of reappraisal that people spontaneously adopt [73]: people with strong DS abilities may find it easier to dismiss their appraisals, whereas those lacking DS ability may fall back on TS, preferring to seek new appraisal frames. Assessment of MC abilities could therefore serve to guide targeted interventions aimed at either (i) improving deficient CER abilities, perhaps via training, or instead (ii) tailoring interventions to capitalize on the existing strengths of each individual.

**Table 1:** Possible Involvement of DS and TS in Emotion Regulation^a^, ^b^

Strategy	Description	DS	TS	Possible role of MC
Expressive suppression [85]	Suppressing behaviors associated with emotion	0	0	None; action stopping likely involves inhibitory control, however
Emotion suppression [101]	DS of emotional reactions	+	0	May typically entrain the suppression of cognitive contents related to emotion
Thought suppression [93]	Suppressing thoughts associated with emotions	+	0	Suppressing emotion-related thoughts; may typically entrain emotion suppression. Unlike cognitive avoidance (below), it entails inhibition of unwanted content to suppress it
Cognitive avoidance [102]	Cognitively avoiding reminders of emotion	0	0	Unlike thought suppression, which forces a person to confront reminders and suppress retrieval of a thought, cognitive avoidance skirts the MC issue by steering clear of reminders
Distraction [103]	Avoiding emotion by focusing on innocuous events	0	+	Mnemonic distraction, in which a person generates diversionary thoughts in response to reminders, is plausibly thought of as a TS phenomenon. Note that this is in contrast to environmental distraction which focuses on external stimuli and events that take attention from the feeling
Reappraisal [72]	Changing the interpretation of emotional events	+	+	Suppressing dominant interpretation; retrieving information to generate a substitute interpretation
Problem solving [91]	Actively engaging with source of emotional distress	0	+	Generating solutions encodes alternative information/thoughts that may act like TS
Worry [104]	Recurrent, intrusive problem-solving of future events	−	+	Poor suppression of emotional thoughts combined with strong substitution of (fruitless) problem-solving thoughts
Rumination [39]	Recurrent, intrusive passive cognition on emotional events	−	0	Poor suppression of emotional thoughts might lead to rumination
Acceptance [91]	Adopting an accepting stance towards emotions	0	0	To the degree that accepting emotions involves not regulating them, no relationship is predicted
Mindfulness [27]	Adopting a non-judgmental stance towards emotion	+	0	To the extent that adopting a non-judgmental stance entails the suppression of negative interpretations of an emotional state, suppression could be involved
Behavioral avoidance [27]	Physically avoiding reminders of emotion	0	0	None, because there is no clear cognitive control component to this strategy

a List adapted from [25].

b Symbols: +, hypothesized positive role of DS/TS; −, hypothesized negative role; 0, no hypothesized role.

Aside from understanding the broad component processes of CER strategies, the MC literature offers novel hypotheses about the neural machinery of emotion regulation. For example, research on MC suggests that CER research should investigate prefrontal influences on the medial temporal lobe (MTL) more broadly, and not simply on the amygdala. If the initial suppression of an appraisal engages DS, for example, hippocampal activity may be suppressed, contributing to successfully reorienting to and encoding a novel appraisal. Similarly, the evidence discussed above that DS efficacy is related to differences in local inhibitory circuits within the hippocampus 58, 60 suggests that there could be a connection between hippocampal GABA and the ability to implement CER techniques that rely on suppression of emotional memories. Parallel GABAergic mechanisms might be important in forms of CER that emphasize amygdala inhibition and the suppression of negative affective states, but might be less important for CER focused on enhancing positive emotion [56].

## Concluding Remarks

Emotion regulation is the ability interrupt or alter the generation of emotional states [19]. Because many emotions we experience in daily life stem from recall of emotionally charged memories, the capacity to control whether and when such recall occurs must be a central emotion-regulation mechanism. In this article we have explored how this mechanism might be employed and support different forms of CER. Although we hope to have offered a convincing argument in support of this perspective, there remains a great deal of work to be done (see Outstanding Questions) to map out how MC supports emotion regulation. For instance, while we believe that MC capacities play a central role in CER, there are several other factors (e.g., working memory capacity, emotional constitution, and executive control abilities aside from inhibition, to name but a few) that influence the capacity to engage in CER. An important topic for future research will be to map out how such factors and MC abilities for both emotional and non-emotional memories interact in determining individual strengths and weaknesses in CER. Importantly, however, we predict that MC will emerge as a key factor because of the central role of memory in emotional processing. Ultimately, therefore, we believe that an understanding of the human ability to surmount the compulsions of our past must be grounded in a mechanistic account of how our memories of that past are controlled.

Outstanding Questions To what degree are the psychological and neural mechanisms supporting affective control coextensive with those supporting general MC?

Can the emotion associated with an event be inhibited without affecting its mnemonic content? Does inhibiting an emotion fundamentally require inhibition of the thoughts and memories that precipitated it?

Do differing approaches to MC vary in whether the forgetting they produce leaves emotional responses intact and free to influence behavior?

Do emotional or high arousal stimuli/memories require special regulatory mechanisms to control, or are they on a continuum with other non-emotional thought contents?

Do MC abilities predict preferences for, and the efficacy of, different emotion-regulation strategies?

Does training someone on MC improve their emotion-regulation ability?

Does reappraisal induce forgetting of stimulus or event details that are not consistent with the new narrative generated?

Are MC abilities similarly important for explicit and implicit forms of emotion regulation?

## Glossary

**Affect-suppression effect**: a combined reduction of intrusiveness and affective responses seen to affective memories following DS efforts.
**Amnesic shadow**: the phenomenon that suppressing a memory of an event induces forgetting not only of the event but also of the events that precede and follow the suppression attempt itself.
**Amygdala**: a collection of nuclei in the medial temporal lobe (MTL) that are important in orchestrating affective reactions.
**Cognitive avoidance**: a coping strategy that involves avoiding processing of reminders of a topic.
**Direct suppression (DS)**: an inhibitory MC process that involves interrupting ongoing retrieval of memories when confronted with a reminder.
**Endogenous emotional reactions**: emotional reactions that are caused by internal sources of information, such as our memories or ongoing streams of thought.
**Episodic prospection**: memory-like episodic simulations of future situations. The term is used because of the similarity in the phenomenological and neural processes of such future-oriented cognition with those of past-oriented memories.
**Exogenously elicited emotions**: emotional states caused by events or stimuli in the external world, such as for example seeing a snake in the grass on a garden path or a surprise encounter with a loved one.
**Expressive suppression (ES)**: an emotion-regulation strategy characterized by inhibiting behavioral expression of emotional reactions.
**Extinction learning**: the gradual decrease of threat conditioned responses seen when a conditioned stimulus is presented without reinforcement.
**GABAergic interneurons**: inhibitory neurons that use γ-aminobutyric acid (GABA) as their chief inhibitory neurotransmitter. They are believed to be involved in the mechanism underlying top-down inhibition of activity in the hippocampus.
**Hippocampus**: a structure in the MTL that plays a key role in episodic memory.
**Memory control (MC)**: the collection of cognitive control processes that enable motivated forgetting.
**Mnemonic emotional material**: memories with emotional connotations, such as memories of traumatic events.
**Post-traumatic stress disorder (PTSD)**: a disorder characterized by the debilitating intrusion into daily life of negatively charged memories following a traumatic event.
**Predictive models**: our expectations about the type of sensory stimuli that are likely to occur, given our previous experience with a given stimulus or situation, or our expectations based on the similarity of a novel situation to a previous situation.
**Reappraisal**: an emotion-regulation strategy aimed at changing what one feels by changing one's interpretation of the external or internal stimuli that caused an emotional state.
**Reinstatement principle**: the idea that suppression of a given memory is neurally implemented by inhibition not only of the hippocampus but also of neural processing in extra-hippocampal (e.g., neocortical) regions that represent the content of the to-be-suppressed memory and that are reinstated by hippocampal retrieval processes.
**Resilience**: the capacity to adaptively weather stress and negative emotional events without long-term consequences.
**Rumination**: in psychology, rumination is the repetitive thinking about past events of an emotional nature, leading to reinstatement and possible amplification of the original emotion. Rumination is frequently associated with psychopathological disturbances of anxiety and depression. Generally thought of as being a maladaptive emotion-regulation strategy where trying to rethink the problems of the past leads to perpetuation of their emotional impact.
**Spontaneous ER/MC**: CER and MC efforts that an individual engages in without direct instruction, also outside the laboratory.
**Thought substitution (TS)**: an MC process that involves avoiding recall of a memory when confronted with a reminder by using the reminder to recall an alternative memory.
**Volitional cognitive emotion regulation (CER)**: the subset of emotion-regulation efforts that involve a conscious goal to use cognitive operations to modulate emotional states (e.g., reappraisal).
